# Normative values of radiographic parameters in coronal plane lower limb alignment in a general Japanese population: A cross‐sectional study in the Iwaki cohort

**DOI:** 10.1002/jeo2.70207

**Published:** 2025-04-01

**Authors:** Ryoto Kura, Eiji Sasaki, Eitaro Sato, Yukiko Sakamoto, Yuka Kimura, Kyota Ishibashi, Eiichi Tsuda, Yasuyuki Ishibashi

**Affiliations:** ^1^ Department of Orthopaedic Surgery, Graduate School of Medicine Hirosaki University Hirosaki Japan; ^2^ Department of Rehabilitation Medicine, Graduate School of Medicine Hirosaki University Hirosaki Japan

**Keywords:** knee osteoarthritis, coronal plane alignment of the knee, general population, cross‐sectional study, lower limb alignment, normative value

## Abstract

**Purpose:**

The purpose of this study was to estimate normative values of radiographic parameters in lower limb alignment and investigate age‐related changes in lower limb alignment in a general Japanese population.

**Methods:**

A total of 1474 knees in 737 volunteers (307 men and 430 women) who participated in the Iwaki cohort study were enroled. Standing anterior‐posterior radiographs were assessed using the Kellgren–Lawrence (KL) grading scale, with KL ≥Grade 2 defined as osteoarthritis (OA). Radiographic parameters measured included the hip–knee–ankle angle (HKAA), femorotibial angle (FTA), mechanical lateral distal femoral angle (mLDFA), medial proximal tibial angle (MPTA), joint line convergence angle (JLCA) and weight‐bearing line ratio (WBLR). Normative values were estimated as the mean ± 2 standard deviations for all participants without knee OA (KOA) aged 18–39 years, and associations with age were analyzed.

**Results:**

Normative values in overall young healthy participants were: HKAA, 1.8 ± 5.1°; FTA, 175.7 ± 6.5°; mLDFA, 86.4 ± 3.4°; MPTA, 85.8 ± 4.1°; JLCA, 1.2 ± 2.7° and WBLR, 39.7 ± 23.7%. The HKAA, FTA, MPTA and WBLR values differed significantly between sexes. In men without KOA, HKAA, FTA, MPTA and WBLR values correlated very weakly with age (*r* = −0.189 to 0.220). In contrast, only HKAA correlated very weakly with age in women without KOA (*r* = 0.096).

**Conclusions:**

Estimated normative values of radiographic parameters in coronal plane lower limb alignment differed significantly between sexes. These data might be useful when considering the aetiology and therapeutic strategy for KOA.

**Level of Evidence:**

Level II.

**Abbreviations**
BMD: bone mineral density
BMI: body mass index
FTA: femorotibial angle
HKAA: hip–knee–ankle angle
ICC: intraclass correlation coefficient
JLCA: joint line convergence angle
KL: Kellgren–Lawrence
KOA: knee osteoarthritis
mLDFA: mechanical lateral distal femoral angle
MPTA: medial proximal tibial angle
OA: osteoarthritis
SD: standard deviation
TKA: total knee arthroplasty
WBLR: weight‐bearing line ratio

## INTRODUCTION

Knee osteoarthritis (KOA) is a common and multifactorial condition causing disability and chronic pain in older individuals [17]. Among various risk factors for KOA, lower limb varus malalignment is considered a strong risk factor for the progression of KOA in combination with osteoporosis [1, 7, 27]. A varus angle >6° increases the risk of cartilage damage, and varus deformity is a precipitating factor for KOA [33]. So‐called constitutional varus, malalignment at the end of growth ≥3° varus, is observed in 32% of young men and 17% of young women. Many individuals with KOA may have a constitutive varus alignment prior to the onset of KOA [3]. The pathogenesis of varus and factors related to progression, however, are unclear.

Currently, lower limb alignment is attracting increased attention in clinical practice. The correction target angle for an osteotomy around the knee joint is still controversial, and constitutional alignment is frequently assessed for determining the therapeutic strategy [2, 4]. For total knee arthroplasty (TKA), coronal plane alignment of the knee classification is used to reproduce the constitutional varus alignment as kinematic alignment TKA [10, 21]. While epidemiological studies using real‐world big data on alignment and phenotype of KOA suggest alignment‐based therapeutic strategies, these data are still based on the patient's data set [9, 26]. These strategies aimed at better clinical outcomes rely on accurate normative parameters, highlighting the importance of understanding the actual normative values of lower limb alignment. However, the normative value and its age‐related changes are not fully revealed, because clarifying them requires large‐sample epidemiologic studies in healthy people, and such studies are scarce.

The purpose of this study was to estimate normative values of lower limb alignment in a young, healthy general population without radiographic osteoarthritic changes from the Iwaki cohort study. Age‐related changes in the normative values and their association with bone mineral density (BMD) were also investigated. We hypothesized that sex, age and BMD contribute to alignment changes that may lead to KOA.

## MATERIALS AND METHODS

### Iwaki cohort and patient recruitment

The present study was approved by the Ethics Committee of the Hirosaki University Graduate School of Medicine (No. 2021‐019H_7) and conducted in accordance with the tenets of the 1964 Helsinki Declaration and its later amendments. All participants volunteered for the Iwaki Health Promotion Project, a community‐based preventative medicine programme that aims to improve the average life expectancy by conducting general health checkups and prophylactic interventions [5, 12, 25]. Written informed consent was obtained from each participant prior to their involvement in the study. Epidemiologic studies of the knee are included in the Iwaki Project, which was started in 2008.

**Figure 1:**
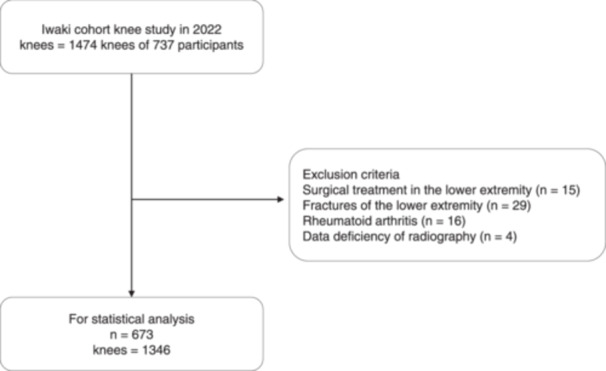
Flowchart illustrating participant inclusion for this study. The participants included in or excluded from the current study are shown.

A total of 737 volunteers (307 men and 430 women) participated in the project in 2022. Of the 737 volunteers, 64 were excluded from this study for the following reasons: hip or knee arthroplasty (*n* = 3); rheumatoid arthritis (*n* = 16); previous intra‐ or extra‐articular fractures of the femur or tibia (*n* = 29); previous knee surgeries, including arthroplasty, osteotomy, anterior cruciate ligament reconstruction (*n* = 12); and radiography nor performed (*n* = 4). Finally, 673 participants (273 men and 400 women) were enroled in the statistical analyses (Figure 1). Participants were divided into five age categories as follows: <40, 40–49, 50–59, 60–69 and ≥70 years.

### Measurement of radiographic parameters

Normative values of radiographic parameters in coronal plane lower limb alignment were defined as the mean value ± 2 standard deviation (SD) of individuals in the <40 age group [22, 23].

**Figure 2:**
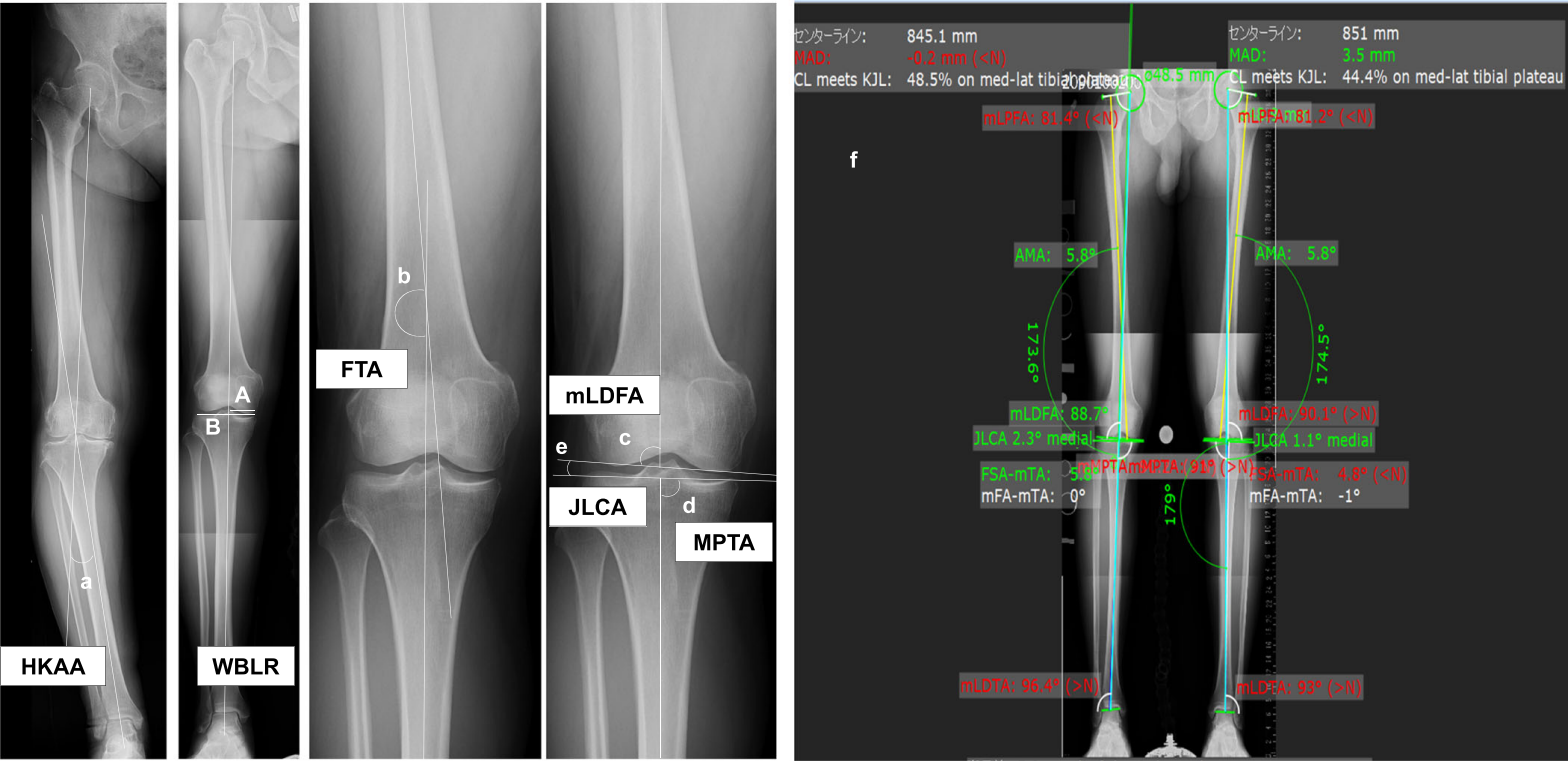
Radiographic parameters in lower limb alignment. HKAA, hip–knee–ankle angle (a), FTA, femorotibial angle (b), mLDFA, mechanical lateral distal femoral angle (c), MPTA, medial proximal tibial angle (d), JLCA, joint line convergence angle (e), WBLR, weight‐bearing line ratio. *The weight‐bearing line ratio was calculated as A/B. (f) Example of measurement using mediCAD.

All participants underwent digital long‐leg radiographs of the lower limb while standing with both patellae facing forward and each knee maximally extended. The Kellgren–Lawrence (KL) grading scale [14] was used to diagnose KOA, and a KL Grade ≥2 was defined as OA. Knee radiographs were imaged using a digital radiography system (CXDI‐40EG, Canon Inc.). Whole‐leg standing radiographs were obtained under the following conditions: distance between the cassette and tube, 300 cm; tube voltage, 85 kV and tube current, 200 mA. Participants stood barefoot in a closed‐leg stance with the patellae facing forward. Measurements of the radiographic parameters of the lower limb alignment were taken with mediCAD® software Version 5.5 (TOYO Corporation) as follows: the hip–knee–ankle angle (HKAA), defined as the angle between the mechanical femoral and mechanical tibial axis [24]; the femorotibial angle (FTA), defined as the lateral angle at which the line through the centre of the femur and the line through the centre of the tibia overlap [24]; the mechanical lateral distal femoral angle (mLDFA), defined as the lateral angle between the mechanical axis and knee joint lines of the femur [18, 24]; the medial proximal tibial angle (MPTA), defined as the medial angle between the mechanical axis and the knee joint lines of the proximal tibia [18, 24]; the joint line convergence angle (JLCA), defined as the angle between the distal femoral joint surface and the proximal tibial joint surface with lateral openings assigned positive values [18, 24]; and the weight‐bearing line ratio (WBLR), defined as the percentage of the tibial proximal joint surface along the Mikulicz line (functional axis of the lower limb connecting the center of the femoral head and the center of the ankle joint) [31] (Figure 2).

Intra‐ and inter‐observer reliability were evaluated by intraclass correlation coefficients (ICCs). To assess inter‐observer reliability, the same 50 radiographs of 100 knees were independently measured by an experienced senior orthopaedic surgeon and a resident orthopaedic surgeon. To assess intra‐observer reliability, these 50 radiographs were measured 1 month later by the same observers. Inter‐observer reliability was specifically evaluated using ICCs. ICC (2,1) was high for all parameters measured, with ICCs >0.75 (range: 0.87–0.99). The ICC was 0.998 (0.997–0.999) for HKAA, 0.977 (0.960–0.986) for FTA, 0.992 (0.985–0.995) for mLDFA, 0.953 (0.918–0.972) for MPTA, 0.870 (0.775–0.924) for JLCA and 0.997 (0.985–0.999) for WBLR.

### BMD

Dual‐energy X‐ray absorptiometry (DCS‐600EXV, Hitachi Aloka Medical) was used to determine the BMD of the forearm on the non‐dominant side at the one third distal radius. In individuals with a history of fracture, the dominant side was measured.

### Statistical analysis

To achieve a statistical power of 80% with an *α* of 0.05 indicating a large effect size (*r* = 0.5), the power analysis revealed that the following sample sizes were required for detecting differences in radiographic assessments using the Mann–Whitney *U* test: HKAA, 103 participants; FTA, 249 participants; mLDFA, 212 participants; MPTA, 88 participants; JLCA, 243 participants and WBLR, 142 participants. Using the SD of the radiographic assessment, the power was >0.9 for all parameters.

Demographic data are expressed as mean ± SD. Due to the non‐normal distribution of some demographic parameters based on the Shapiro–Wilk test, the Mann–Whitney *U* test was used for continuous variables to compare the demographic data and radiographic parameters between men and women. Spearman's rank correlation was used to assess the correlations among radiographic parameters, age, body mass index (BMI) and BMD. Radiographic parameters among age categories were compared by analysis of variance and Tukey's test as post hoc analysis in men and women, respectively. Finally, to investigate factors related to radiographic parameters among participants with and without KOA, regression analysis was performed with HKAA, FTA, mLDFA and MPTA as dependent variables; and age, sex, BMI and BMD as independent variables. The statistical analysis was performed using IBM SPSS Statistics version 29 (IBM). *p* < 0.05 was considered to indicate statistical significance.

## RESULTS

### Demographics

The overall prevalence of radiologic KOA was 31.2%; 18.7% in men and 39.8% in women (*p* < 0.001). Smoking and drinking habits were significantly more prevalent in men (*p* < 0.001; Table 1). Both BMI and BMD were significantly greater in men (*p* < 0.001).

**Table 1:** Demographic data of the participants.

	Overall	Men	Women	*p* value
Number, people	673	273	400	–
Age, years	52.6 ± 15.0	53.8 ± 15.0	51.8 ± 14.9	0.094
Body mass index, kg/m^2^	23.0 ± 3.3	24.0 ± 3.1	22.4 ± 3.3	<0.001**
Bone mineral density, g/cm^2^	0.68 ± 0.11	0.76 ± 0.068	0.62 ± 0.098	<0.001**
KL Grades 0 and 1, %	926 (68.8%)	444 (81.3%)	482 (60.2%)	<0.001**
KL Grade 2, %	328 (24.4%)	72 (13.2%)	256 (32.0%)	<0.001**
KL Grade 3, %	88 (6.50%)	30 (5.5%)	58 (7.30%)	0.201
KL Grade 4, %	4 (0.30%)	0 (0%)	4 (0.50%)	0.098
Smoking habit, %	113 (16.8%)	75 (27.5%)	38 (9.50%)	<0.001**
Drinking habit, %	344 (51.1%)	184 (67.4%)	160 (40.0%)	<0.001**
Fitness habit, %	180 (26.7%)	73 (26.7%)	107 (26.8%)	0.998

*Note*: Mean and standard deviation of age, body mass index and bone mineral density were compared using the Mann–Whitney *U* test. *p* < 0.05 was considered significant.

Abbreviation: KL, Kellgren–Lawrence.

* *p* < 0.05.

** *p* < 0.01.

### Radiographic parameters in lower limb alignment in overall participants

Significant differences in HKA, FTA, mLDFA, MPTA, JLCA and WBLR were detected between men and women without KOA. Across overall participants, men had significantly larger HKAA, FTA and mLDFA, and smaller MPTA, JLCA and WBLR than women (*p* < 0.001; Table 2).

**Table 2:** Lower limb alignment parameters of all participants.

	Non‐KOA			KOA			Overall			
	Men	Women	*p* value	Men	Women	*p* value	Total	Men	Women	*p* value
Number, knees	444	482	–	102	318		1346	546	800	
HKAA, degree	2.68 ± 2.5	1.69 ± 2.6	<0.001**	2.62 ± 2.8	2.26 ± 2.8	0.134	2.22 ± 5.2	2.68 ± 2.4	1.95 ± 2.6	<0.001**
FTA, degree	176.1 ± 3.3	175.2 ± 3.4	<0.001**	175.6 ± 3.7	175.6 ± 3.6	0.647	175.6 ± 6.8	176.1 ± 3.3	175.6 ± 3.5	<0.001**
mLDFA, degree	86.9 ± 1.8	86.5 ± 1.8	<0.001**	86.7 ± 1.6	86.9 ± 2.1	0.506	86.7 ± 3.8	87.0 ± 1.7	86.6 ± 1.9	0.003*
MPTA, degree	85.4 ± 1.9	86.2 ± 2.0	<0.001**	85.8 ± 2.0	86.5 ± 2.0	0.001*	86.0 ± 4.0	85.4 ± 2.0	86.3 ± 1.9	<0.001**
JLCA, degree	1.10 ± 1.2	1.43 ± 1.4	<0.001**	1.79 ± 1.4	1.88 ± 1.5	0.641	1.45 ± 2.8	1.1 ± 1.3	1.6 ± 1.5	<0.001**
WBLR, degree	36.5 ± 10.5	40.3 ± 11.8	<0.001**	37.2 ± 11.9	38.0 ± 12.3	0.383	38.3 ± 23.2	36.6 ± 10.6	38.0 ± 12.0	<0.001**

*Note*: Mean + standard deviation of radiographic lower extremity alignment parameters were compared using the Mann–Whitney *U* test. *p* < 0.05 was considered significant.

Abbreviations: FTA, femorotibial angle; HKAA, hip–knee–ankle angle; JLCA, joint line convergence angle; KOA, knee osteoarthritis; mLDFA, mechanical lateral distal femoral angle; MPTA, medial proximal tibial angle; WBLR, weight‐bearing line ratio.

* *p* < 0.05.

** *p* < 0.01.

### Normative values of radiographic parameters in lower limb alignment

Normative values (mean ± 2 SD) in young men were as follows: HKAA, 2.85 ± 5.0°; FTA, 176.8 ± 6.2°; mLDFA, 86.7 ± 3.8°; MPTA, 84.9 ± 4.0°; JLCA, 1.11 ± 2.4°; and WBLR, 35.2 ± 22.0%. In women, normative values were: HKAA, 1.19 ± 5.0°; FTA, 175.0 ± 6.4°; mLDFA, 86.3 ± 3.2°; MPTA, 86.3 ± 3.8°; JLCA, 1.26 ± 2.8°; and WBLR, 42.3 ± 23.2%. Significant differences were detected between men and women in all radiographic parameters except mLDFA and JLCA (Table 3).

**Table 3:** Normative values of lower limb alignment for each age group between men and women without KOA.

	Men						Women					
	<40 Normative values	40–49	50–59	60–69	>70	*p* value	<40 Normative values	40–49	50–59	60–69	>70	*p* value
Number	98	137	63	84	62	–	169	134	80	68	31	–
HKAA, degree (1st and 3rd quartiles) (95% CI)	2.85 ± 5.0 (0.88–4.7) (2.36–3.35)	2.99 ± 4.1 (1.65–4.45) (2.64–3.33)	3.27 ± 3.9 (1.80–4.90) (2.78–3.76)	2.04 ± 6.0 (0.13–4.23) (1.39–2.69)	2.00 ± 5.3 (0.75–3.52) (1.33–2.67)	0.004*	1.19 ± 5.0 (−0.50 to 3.20) (0.81–1.56)	2.05 ± 5.1 (0.25–3.83) (1.62–2.48)	2.14 ± 5.0 (0.50–3.80) (1.58–2.69)	1.87 ± 5.5 (0.13–3.60) (1.20–2.53)	1.36 ± 5.5 (−0.50 to 3.50) (0.36–2.36)	0.013*
FTA, degree (1st and 3rd quartiles) (95% CI)	176.8 ± 6.2 (174.7–180.0) (176.2–177.4)	176.4 ± 5.9 (174.5–177.6) (175.9–176.9)	176.8 ± 5.7 (175.3–177.9) (176.1–177.5)	175.0 ± 7.3 (172.7–176.6) (174.2–175.8)	174.9 ± 7.3 (172.7–177.5) (174.0–175.8)	<0.001**	175.0 ± 6.4 (172.8–176.6) (174.5–175.5)	175.7 ± 6.8 (173.5–177.0) (175.1–176.3)	175.7 ± 6.4 (173.7–177.1) (175.0–176.4)	174.9 ± 7.2 (172.7–176.5) (174.0–175.8)	174.2 ± 7.4 (171.5–176.5) (172.9–175.6)	0.077
mLDFA, degree (1st and 3rd quartiles) (95% CI)	86.7 ± 3.8 (85.4–87.9) (86.3–87.1)	87.1 ± 3.4 (86.1–88.3) (86.8–87.4)	87.4 ± 3.1 (86.4–88.4) (87.0–87.8)	86.5 ± 3.6 (85.3–87.6) (86.1–86.9)	87.1 ± 3.8 (85.7–88.5) (86.6–67.5)	0.008*	86.3 ± 3.2 (85.2–87.3) (86.0–86.5)	86.7 ± 3.7 (85.6–87.7) (86.4–87.0)	86.7 ± 3.4 (85.6–87.8) (86.3–87.1)	86.5 ± 4.0 (85.2–87.9) (86.0–87.0)	85.7 ± 4.1 (84.2–87.5) (84.9–86.4)	0.018*
MPTA, degree (1st and 3rd quartiles) (95% CI)	84.9 ± 4.0 (83.8–86.1) (84.5–85.3)	85.4 ± 3.6 (84.4–86.6) (85.1–85.7)	85.0 ± 3.5 (83.9–86.2) (84.6–85.5)	85.6 ± 4.1 (84.3–86.9) (85.1–86.0)	86.0 ± 3.9 (84.9–87.2) (85.5–86.5)	0.005*	86.3 ± 3.8 (84.9–87.9) (86.0–86.6)	86.2 ± 4.1 (84.8–87.7) (85.8–86.5)	86.1 ± 3.7 (85.1–87.2) (85.6–86.5)	86.1 ± 4.3 (84.8–87.3) (85.5–86.6)	86.1 ± 3.2 (85.3–87.2) (85.5–86.7)	0.783
JLCA, degree (1st and 3rd quartiles) (95% CI)	1.11 ± 2.4 (0.20–1.90) (0.87–1.35)	1.24 ± 2.5 (0.30–2.00) (1.03–1.46)	0.88 ± 2.3 (0.10–1.60) (0.59–1.17)	1.12 ± 2.2 (0.30–1.78) (0.88–1.35)	0.95 ± 2.3 (0.10–1.53) (0.66–1.24)	0.274	1.26 ± 2.8 (0.40–2.10) (1.04–1.47)	1.53 ± 3.1 (0.40–2.55) (1.26–1.79)	1.47 ± 2.5 (0.70–2.00) (1.19–1.75)	1.45 ± 2.5 (0.60–2.28) (1.15–1.76)	1.79 ± 2.5 (0.70–2.40) (1.33–2.25)	0.261
WBLR, degree (1st and 3rd quartiles) (95% CI)	35.2 ± 22.0 (26.9–44.1) (33.0–37.4)	34.9 ± 17.9 (28.6–40.7) (33.4–36.4)	34.1 ± 16.2 (28.1–39.2) (32.0–36.1)	39.9 ± 24.6 (31.3–47.5) (37.2–42.5)	40.5 ± 21.2 (34.4–45.6) (37.8–43.2)	<0.001**	42.3 ± 23.2 (33.2–49.9) (40.6–44.1)	38.3 ± 23.4 (29.8–46.7) (36.3–40.3)	38.5 ± 22.6 (30.5–45.9) (36.0–41.0)	40.1 ± 24.6 (32.0–48.3) (37.1–43.0)	42.6 ± 23.4 (34.3–49.8) (38.4–46.9)	0.018*

*Note*: Mean + 2 standard deviations of radiographic lower extremity alignment parameters were compared using Tukey's test. *p* < 0.05 was considered significant.

Abbreviations: FTA, femorotibial angle; HKAA, hip–knee–ankle angle; JLCA, joint line convergence angle; KOA, knee osteoarthritis; mLDFA, mechanical lateral distal femoral angle; MPTA, medial proximal tibial angle; WBLR, weight‐bearing line ratio.

* *p* < 0.05.

** *p* < 0.01.

### Age‐related changes in radiographic parameters of lower limb alignment

Among participants without KOA, HKAA (*p* = 0.004), FTA (*p* < 0.001), mLDFA (*p* = 0.008), MPTA (*p* = 0.005) and WBLR (*p* < 0.001) showed significant age‐related changes in men (Table 3). In women, HKAA (*p* = 0.013), mLDFA (*p* = 0.018) and WBLR (*p* = 0.018) showed significant age‐related changes. In contrast, in men with KOA, no significant age‐related differences in any radiologic parameter were detected. In women with KOA, HKAA (*p* = 0.001), FTA (*p* < 0.001), MPTA (*p* = 0.014), JLCA (*p* < 0.001) and WBLR (*p* = 0.007) showed significant age‐related changes (Table 4).

**Table 4:** Radiographic parameters of lower limb alignment for each age group between men and women with KOA.

	Men						Women					
	<40	40–49	50–59	60–69	>70	*p* value	<40	40–49	50–59	60–69	>70	*p* value
Number	8	9	7	44	34	–	31	42	64	110	71	–
HKAA, degree (1st and 3rd quartiles) (95% CI)	1.49 ± 2.02 (−0.18 to 3.15) (−0.20 to 3.18)	3.13 ± 3.05 (0.25–5.65) (0.79–5.48)	3.09 ± 1.03 (2.00–3.80) (2.13–4.04)	2.46 ± 2.80 (0.33–4.58) (1.61–3.31)	2.85 ± 3.02 (1.60–4.83) (1.80–3.91)	0.697	1.88 ± 2.17 (1.10–3.30) (1.09–2.68)	1.10 ± 2.65 (−0.88 to 3.40) (0.28–1.93)	1.76 ± 2.70 (0.13–3.40) (1.08–2.43)	2.30 ± 2.27 (0.58–3.53) (1.87–2.73)	3.51 ± 3.29 (1.10–6.30) (2.73–4.29)	0.001*
FTA, degree (1st and 3rd quartiles) (95% CI)	175.0 ± 3.83 (171.8–178.1) (171.8–178.2)	176.7 ± 4.29 (173.9–180.5) (173.4–180.0)	176.6 ± 3.17 (174.7–178.0) (173.7–179.6)	175.4 ± 3.67 (172.9–177.2) (174.3–176.5)	175.5 ± 3.94 (173.9–177.5) (174.1–176.9)	0.820	175.9 ± 3.37 (173.5–177.9) (174.7–177.2)	174.0 ± 2.18 (172.4–175.6) (173.3–174.8)	174.9 ± 3.81 (172.5–176.6) (174.0–175.9)	175.6 ± 3.41 (173.6–177.0) (175.0–176.3)	177.1 ± 3.86 (174.5–180.7) (176.2–178.0)	<0.001**
mLDFA, degree (1st and 3rd quartiles) (95% CI)	86.2 ± 1.55 (85.1–87.6) (84.9–87.5)	86.9 ± 2.13 (85.5–88.3) (85.3–88.6)	87.3 ± 2.11 (86.1–88.6) (85.4–89.3)	86.6 ± 1.26 (85.7–87.2) (86.2–87.0)	86.6 ± 1.70 (85.4–87.5) (86.0–87.2)	0.689	86.9 ± 2.01 (85.2–88.3) (86.1–87.6)	87.2 ± 2.06 (85.9–88.2) (86.5–87.8)	87.1 ± 2.28 (85.9–88.4) (86.6–87.7)	86.5 ± 1.76 (85.3–87.7) (86.1–86.8)	87.0 ± 2.46 (85.1–88.4) (86.4–87.6)	0.182
MPTA, degree (1st and 3rd quartiles) (95% CI)	86.0 ± 2.23 (84.6–87.8) (84.1–87.9)	85.4 ± 2.41 (83.3–86.6) (83.5–87.2)	85.6 ± 1.98 (85.0–87.0) (83.8–87.5)	85.9 ± 2.19 (84.4–87.5) (85.2–86.6)	85.8 ± 1.74 (84.4–86.8) (85.2–86.4)	0.965	86.7 ± 1.83 (85.0–88.1) (86.0–87.4)	86.9 ± 1.64 (85.5–88.2) (86.4–87.4)	87.0 ± 1.82 (85.7–88.2) (86.6–87.5)	86.1 ± 2.07 (84.9–87.4) (85.7–86.5)	86.2 ± 2.20 (85.1–87.8) (85.7–86.7)	0.014*
JLCA, degree (1st and 3rd quartiles) (95% CI)	1.31 ± 0.98 (0.35–2.00) (0.49–2.14)	1.64 ± 1.32 (0.85–3.10) (0.63–2.66)	1.64 ± 1.32 (−0.20 to 2.10) (0.34–2.43)	1.39 ± 1.13 (0.60–2.68) (1.31–2.14)	2.09 ± 1.70 (0.68–3.18) (1.50–2.69)	0.547	1.75 ± 1.26 (0.90–2.40) (1.28–2.21)	0.83 ± 1.80 (−0.45 to 2.03) (0.27–1.39)	1.63 ± 1.57 (0.60–2.48) (1.24–2.02)	1.92 ± 1.29 (0.80–2.80) (1.68–2.17)	2.70 ± 1.40 (1.70–3.70) (2.36–3.03)	<0.001**
WBLR, degree (1st and 3rd quartiles) (95% CI)	41.8 ± 8.9 (35.0–49.5) (34.4–49.2)	34.6 ± 12.8 (24.6–46.9) (24.8–44.4)	34.5 ± 4.94 (30.5–40.1) (30.0–39.1)	37.7 ± 12.1 (28.7–46.7) (34.0–41.3)	36.7 ± 13.2 (28.9–41.7) (32.1–41.3)	0.726	39.1 ± 10.7 (32.8–42.9) (35.2–43.0)	42.4 ± 12.5 (32.9–50.8) (38.5–46.3)	39.7 ± 12.3 (33.7–46.5) (36.6–42.8)	39.7 ± 12.3 (32.6–45.8) (36.2–40.0)	38.1 ± 10.2 (22.4–43.6) (29.7–36.5)	0.007

*Note*: Mean + standard deviation of radiographic lower extremity alignment parameters were compared using Tukey's test. *p* < 0.05 was considered significant.

Abbreviations: FTA, femorotibial angle; HKAA, hip–knee–ankle angle; JLCA, joint line convergence angle; KOA, knee osteoarthritis; mLDFA, mechanical lateral distal femoral angle; MPTA, medial proximal tibial angle; WBLR, weight‐bearing line ratio.

* *p* < 0.05.

** *p* < 0.01.

### Correlation among age, BMI, BMD and radiographic parameters

Correlation analyses in participants without KOA revealed that both age and BMD correlated with HKAA, FTA, MPTA and WBLR, but the correlation coefficients were very low (*r* = −0.220 to 0.189) in men (Table 5). In women, HKAA correlated with age, and HKAA and MPTA correlated with BMD, but the correlation coefficients were also very low *(r* = −0.054 to 0.096). For participants with KOA, the correlations among age, BMI, BMD and radiographic parameters were very low (Table 6). Regression analysis in participants without KOA showed that sex was significantly related to HKAA (*p* < 0.001), FTA (*p* = 0.003), mLDFA (*p* = 0.043) and MPTA (*p* < 0.001; Table 7). Age correlated with only FTA (*p* = 0.002) and MPTA (*p* = 0.046). In contrast, BMD significantly correlated with FTA (*p* < 0.001) in the participants with KOA, and sex correlated with both FTA (*p* = 0.042) and MPTA (*p* = 0.002; Table 8).

**Table 5:** Correlation between radiographic parameters and age, BMI and BMD in participants without KOA.

	Men						Women					
	Age		BMI		BMD		Age		BMI		BMD	
Dependent variable	*r*	*p* value	*r*	*p* value	*r*	*p* value	*r*	*p* value	*r*	*p* value	*r*	*p* value
HKAA	−0.139	0.003	**−**0.079	0.098	0.106	0.026*	0.096	0.035*	**−**0.034	0.453	−0.054	0.024*
FTA	−0.220	<0.001**	−0.058	0.025*	0.111	0.020*	**−**0.010	0.824	**−**0.042	0.358	0.020	0.659
mLDFA	0.004	0.927	**−**0.027	0.573	0.025	0.599	0.060	0.889	0.073	0.110	0.039	0.393
MPTA	0.175	<0.001**	0.088	0.063	−0.102	0.031*	**−**0.073	0.110	0.061	0.179	0.092	0.043*
JLCA	**−**0.032	0.496	**−**0.079	0.098	0.020	0.637	0.058	0.206	**−**0.076	0.097	**−**0.037	0.419
WBLR	0.189	<0.001**	0.096	0.043*	−0.121	0.010*	**−**0.070	0.125	0.045	0.322	0.027	0.549

*Note*: Correlations between radiographic parameters and age, BMI and BMD were compared using Spearman's rank correlation. *p* < 0.05 was considered significant.

Abbreviations: BMD, body mineral density; BMI, body mass index; FTA, femorotibial angle; HKAA, hip–knee–ankle angle; JLCA, joint line convergence angle; KOA, knee osteoarthritis; mLDFA, mechanical lateral distal femoral angle; MPTA, medial proximal tibial angle; WBLR, weight‐bearing line ratio.

* *p* < 0.05.

** *p* < 0.01.

**Table 6:** Correlation between radiographic parameters and age, BMI and BMD in participants with KOA.

	Men						Women					
	Age		BMI		BMD		Age		BMI		BMD	
Dependent variable	*r*	*p* value	*r*	*p* value	*r*	*p* value	*r*	*p* value	*r*	*p* value	*r*	*p* value
HKAA	0.076	0.446	0.036	0.722	0.024	0.807	0.216	<0.001**	0.050	0.377	−0.203	<0.001**
FTA	**−**0.053	0.598	0.081	0.418	0.078	0.433	0.190	<0.001**	0.039	0.491	−0.240	<0.001**
mLDFA	0.009	0.926	−0.248	0.012*	**−**0.087	0.383	**−**0.061	0.274	0.016	0.772	0.021	0.710
MPTA	0.0.34	0.737	**−**0.075	0.452	**−**0.063	0.527	−0.123	0.028*	0.059	0.291	0.146	0.009*
JLCA	0.117	0.243	0.202	0.042	**−**0.044	0.660	0.301	<0.001**	0.112	0.045*	−0.191	<0.001**
WBLR	**−**0.033	0.745	**−**0.050	0.621	**−**0.030	0.764	−0.181	<0.001**	**−**0.038	0.504	0.177	0.002*

*Note*: Correlations between radiographic parameters and age, BMI and BMD were compared using Spearman's rank correlation. *p* < 0.05 was considered significant.

Abbreviations: BMD, body mineral density; BMI, body mass index; FTA, femorotibial angle; HKAA, hip–knee–ankle angle; JLCA, joint line convergence angle; KOA, knee osteoarthritis; mLDFA, mechanical lateral distal femoral angle; MPTA, medial proximal tibial angle; WBLR, weight‐bearing line ratio.

* *p* < 0.05.

** *p* < 0.01.

**Table 7:** Regression analysis of age, BMI, BMD and radiographic parameters in lower limb alignment in participants without KOA.

	HKAA		FTA		mLDFA		MPTA	
	*B* (95% CI)	*p* value	*B* (95% CI)	*p* value	*B* (95% CI)	*p* value	*B* (95% CI)	*p* value
Age	−0.01 (−0.02 to 0.007)	0.329	−0.03 (−0.05 to −0.01)	0.002*	0.01 (−0.007 to 0.01)	0.632	0.01 (0–0.02)	0.046*
Sex	−1.09 (−1.56 to −0.61)	<0.001**	−0.94 (−1.57 to −0.32)	0.003*	−0.35 (−0.69 to −0.01)	0.043*	1.05 (0.69–1.42)	<0.001**
BMI	−0.05 (−0.10 to 0.004)	0.072	−0.04 (−0.10 to 0.03)	0.307	−0.01 (−0.05 to −0.03)	0.610	0.03 (−0.02 to 0.07)	0.212
BMD	0.17 (−2.37 to 2.70)	0.899	0.74 (−2.60 to 4.07)	0.665	1.18 (−0.62 to 2.98)	0.199	0.99 (−0.98 to 2.96)	0.322

*Note*: Statistical analysis—logistic regression analysis. Dependent variables—HKAA, FTA, LDFA and MPTA. Independent variables—age, sex, BMI and BMD.

Abbreviations: BMD, bone mineral density; BMI, body mass index; CI, confidence interval; FTA, femorotibial angle; HKAA, hip–knee–ankle angle; KOA, knee osteoarthritis; mLDFA, mechanical lateral distal femoral angle; MPTA, medial proximal tibial angle; OR, odds ratio.

* *p* < 0.05.

** *p* < 0.01.

**Table 8:** Regression analysis of age, BMI, BMD and radiographic parameters in lower limb alignment in participants with KOA.

	HKAA		FTA		mLDFA		MPTA	
	*B* (95% CI)	*p* value	*B* (95% CI)	*p* value	*B* (95% CI)	*p* value	*B* (95% CI)	*p* value
Age	0.02 (−0.005 to 0.05)	0.115	−0.009 (−0.04 to 0.02)	0.579	−0.01 (−0.03 to 0.01)	0.465	−0.01 (−0.02 to 0.02)	0.890
Sex	−0.70 (−1.59 to 0.18)	0.120	−1.21 (−2.37 to −0.04)	0.042*	0.10 (−0.53 to 0.75)	0.762	1.03 (0.38–1.69)	0.002*
BMI	0.03 (−0.05 to 0.11)	0.417	0.07 (−0.03 to 0.18)	0.169	0.01 (−0.05 to 0.06)	0.896	0.001 (−0.06 to 0.06)	0.963
BMD	−2.74 (−1.39 to 8.06)	0.111	−7.56 (−11.99 to −3.14)	<0.001**	−0.46 (−2.94 to 2.02)	0.716	2.22 (−0.27 to 4.71)	0.080

*Note*: Statistical analysis—logistic regression analysis. Dependent variables—HKAA, FTA, LDFA and MPTA. Independent variables—age, sex, BMI and BMD.

Abbreviations: BMD, bone mineral density; BMI, body mass index; CI, confidence interval; FTA, femorotibial angle; HKAA, hip–knee–ankle angle; KOA, knee osteoarthritis; mLDFA, mechanical lateral distal femoral angle; MPTA, medial proximal tibial angle; OR, odds ratio.

* *p* < 0.05.

** *p* < 0.01.

## DISCUSSION

In this study, normative values of lower limb coronal plane alignment were estimated from a large‐sample cohort study in the general population. The parameters differed between men and women, even among participants without radiographic KOA. These parameters of lower limb alignment were not affected by age, BMI or BMD in participants without KOA, which demonstrate these normative values are real healthy constitutional values that have not been influenced by age or bone metabolism. And these results suggest that constitutional alignment is unique to each individual. Individuals in the normative range of lower limb alignment tend to maintain healthy joints compared with the general young, healthy population unless they are affected by trauma, osteoporosis, or other metabolic conditions. On the other hand, deviations from the normative range of lower limb alignment may be related to the progression of KOA and serve as reference data for researching the aetiology of KOA progression. Also, in considering the osteotomy around the knee, the information about the centre of deformity or its age‐related changes is important. Furthermore, with the recent movement of applying coronal plane alignment of the knee classification on knee arthroplasty surgery, the normative value regarding the real degree of constitutional varus should be supplied because these data would be used for the target angle for surgical planning. So, the authors believe these data would be helpful for orthopaedic surgeons.

The present study revealed normative values of various parameters in the Japanese general population. Similar studies have been conducted in other countries. The HKAA in young healthy subjects in Europe and the United States is approximately 1.3°, and approximately 32% of men have a constitutional varus alignment [3]. In young healthy Asians (Indians and Koreans), however, HKAA is approximately 2.4°, indicating that they have more varus malalignment than Westerners [28]. In addition, the HKAA of young healthy Japanese is reported to be 2.3° [29]. We found that HKAA was 2.2° with a 95% confidence interval ranging from 2.0° to 2.3°, indicating that healthy Japanese participants had a varus malalignment. mLDFA and MPTA are reported to be 87.9° and 87.0° in Belgium, respectively, and 87.3° and 85.8° in Asians, respectively [3, 28]. In this study, mLDFA and MPTA were 86.4° and 85.8°, respectively, and MPTA showed a more medial alignment compared with that in Westerners. Asians may exhibit a greater degree of varus malalignment compared with those in other countries due to lifestyle habits such as sitting on the floor or sitting cross‐legged, or to occupations such as farming [8].

In the present study, sex differences in all radiologic parameters were found in healthy subjects. Consistent with previous reports, our results showed a greater tendency towards varus alignment in men. A large survey of healthy Korean subjects also reported significantly greater varus alignment in men [11]. In the present study, the MPTA was 84.9° in young healthy men, which is a smaller value than that in the reports mentioned above. The relationship between young men and constitutional varus alignment may indicate that intense sports activity during growth contributes to the development of varus knee [30]. Notably, however, we did not find significant differences between sexes in exercise habits across all participants. It is also possible that the smaller MPTA in men contributes to their greater varus alignment compared with that in women [13, 15].

Significant age‐related differences in lower limb alignment were observed in all radiologic parameters except JLCA in men without KOA, but not in any parameters among all participants with KOA. In women with KOA, significant differences were detected between age groups for many radiologic parameters, except mLDFA (Tables 3 and 4). There are several possible reasons for the differences in radiologic parameters by age between men and women with and without KOA. HKAA increases with the progression of KOA, with MPTA being the greatest contributing factor [7]. Therefore, we hypothesized that younger men with smaller MPTA would develop KOA with increasing age, while older men with a larger MPTA would not develop KOA, resulting in differences in alignment between age groups. In women, age correlated with KOA. Age‐related changes in coronal plane alignment may be more pronounced in women. The present study was a cross‐sectional study, however, and long‐term longitudinal studies are needed to investigate this further.

This study also investigated the relationship between BMD and alignment. BMD is reported to be significantly lower in the femoral neck of KOA knees compared with non‐osteoarthritic knees, with no difference in alignment [6]. It is also reported that BMD is related to varus deformity in postmenopausal women [32]. We found a negative correlation between MPTA and BMD in men without KOA. It may be that MPTA tends to be smaller in younger patients with a higher BMD. In women with KOA, on the other hand, MPTA positively correlated with BMD. This may indicate that osteoporosis causes wear and tear of the bone structure in advanced KOA, exacerbating joint deformity [20]. Regression analysis results show that BMD is significantly associated with FTA in individuals with KOA, suggesting that BMD is involved in lower limb alignment.

Normal lower limb alignment has recently attracted increasing attention, and the TKA technique has undergone significant changes. The concept of a custom‐fit TKA, proposed by Howell et al. in 2008, evolved into a kinematic alignment TKA, which has since become popular [16, 19]. Kinematic alignment TKA is believed to improve patient satisfaction by recreating constitutional alignment, resulting in natural knee motion and good soft tissue balance. Therefore, it is important to evaluate the constitutional alignment of each individual patient. Here, we present the radiologic parameters of healthy adults in each age group, although our participants were all Asian. Our findings will facilitate the estimation of innate alignment during preoperative planning for TKA and high tibial osteotomy procedures.

## LIMITATIONS

This study has several limitations. First, this study used a two‐dimensional assessment of lower limb alignment using X‐ray long lower limb radiographs. We did not consider lower limb rotation and knee joint flexion contracture, which may also influence measurements of these parameters. Second, we did not investigate femoral bowing, and therefore, alignment changes due to bowing deformity could not be considered. Third, this study was based on standard values of lower limb alignment in one region of Japan, not necessarily the standard values of lower limb alignment for the entire adult Japanese population. Fourth, because this was a cross‐sectional study, we cannot conclude from the results of this study that lower limb alignment changes with age. Fifth, the forearm was used to measure BMD. Although the hip or spine BMD is considered more indicative of bone health, these measurements require a large facility to shield radioactive materials. Our measurements were obtained in the limited space of a public hall as a part of a community‐based general health check project. Finally, in these large sample population‐based studies, the results should be carefully interpreted because of their effect size, and it is hard to translate to the population level.

Despite these limitations, our findings provide valuable information on constitutional lower limb alignment prior to TKA surgery and osteotomy.

## CONCLUSIONS

Normative values of radiographic parameters in coronal plane lower limb alignment in the Japanese general population were shown. MPTA‐based constitutional varus was observed in men and women, and it was not correlated with age, BMI or BMD. These normative values of radiographic parameters could be used to consider indications or therapeutic strategies for osteotomy around the knee and arthroplasty surgery.

## AUTHOR CONTRIBUTIONS

Eiji Sasaki was responsible for the organization and coordination of this study, and was the chief investigator and responsible for the data analysis. Ryoto Kura, Eitaro Sato, Yukiko Sakamoto, Yuka Kimura, Kyota Ishibashi, Eiichi Tsuda and Yasuyuki Ishibashi developed this study design. All authors contributed to the writing of the final manuscript.

## CONFLICT OF INTEREST STATEMENT

The authors declare no conflicts of interest.

## ETHICS STATEMENT

The study was approved by the Ethics Committee of Hirosaki University Graduate School of Medicine (No. 2021‐019H_7) and conducted in accordance with the 1964 Helsinki Declaration and its later amendments or comparable ethical standards. All participants provided written informed consent before participation.

## Data Availability

The data sets generated during and/or analyzed during the current study are available from the corresponding author on reasonable request.
